# The Response Dynamics and Function of Cholinergic and GABAergic Neurons in the Basal Forebrain During Olfactory Learning

**DOI:** 10.3389/fncel.2022.911439

**Published:** 2022-07-27

**Authors:** Ping Zhou, Penglai Liu, Ying Zhang, Dejuan Wang, Anan Li

**Affiliations:** Jiangsu Key Laboratory of Brain Disease and Bioinformation, Research Center for Biochemistry and Molecular Biology, Xuzhou Medical University, Xuzhou, China

**Keywords:** basal forebrain, cholinergic neuron, GABAergic neuron, fiber photometry, go/no-go

## Abstract

Modulation of neural circuits is essential for flexible sensory perception and decision-making in a changing environment. Cholinergic and GABAergic projections to the olfactory system from the horizontal limb of the diagonal band of Broca (HDB) in the basal forebrain are crucial for odor detection and olfactory learning. Although studies have demonstrated that HDB neurons respond during olfactory learning, how cholinergic and GABAergic neurons differ in their response dynamics and roles in olfactory learning remains unclear. In this study, we examined the response profiles of these two subpopulations of neurons during passive odor exposure and associative olfactory learning. We show that the excitatory responses in both cholinergic and GABAergic neurons tended to habituate during repeated passive odor exposure. However, while these habituated responses were also observed in GABAergic neurons during a go-go task, there was no such habituation in cholinergic neurons. Moreover, the responses to S+ and S− trials diverged in cholinergic neurons once mice learned a go/no-go task. Furthermore, the chemogenetic inactivation of cholinergic neurons in the HDB impaired odor discrimination. Together, these findings suggest that cholinergic neurons in the HDB reflect attention to positive reinforcement and may regulate odor discrimination *via* top–down inputs to the olfactory system.

## Introduction

Acetylcholine is a crucial neuromodulator of brain function. It originates predominantly from cholinergic neurons located in the basal forebrain, which also contains GABAergic and glutamatergic neurons (Gritti et al., 2006; Do et al., 2016). As one of the most important and widely projecting neuromodulatory centers in the mammalian brain, the basal forebrain has been implicated in sensory perception, attention, arousal, and learning and memory (Herrero et al., 2008; Conner et al., 2010; Robinson et al., 2011; Chubykin et al., 2013; Froemke et al., 2013; Han et al., 2014).

The olfactory system is an important sensory system for mammals, and olfactory centers such as the olfactory bulb and the piriform cortex receive dense innervation from the basal forebrain (Luskin and Price, 1982; De Saint Jan, 2022). Both cholinergic and GABAergic afferent fibers from the horizontal limb of the diagonal band of Broca (HDB), a sub-region of the basal forebrain, form dense innervations with olfactory centers (Zaborszky et al., 1986; Villar et al., 2021; De Saint Jan, 2022). For example, cholinergic axons innervate multiple layers of the olfactory bulb and acetylcholine receptors are widely expressed in the olfactory bulb and the piriform cortex (Mechawar et al., 2004; Gomez et al., 2005; Saar et al., 2007; Smith et al., 2015; Case et al., 2017). Similarly, HDB GABAergic inputs form functional connections with neurons in the olfactory bulb (Nunez-Parra et al., 2013; Sanz Diez et al., 2019). Therefore, it is not unexpected that the cholinergic/GABAergic projections from the HDB to olfactory centers modulate olfactory processing and olfactory behaviors (Linster et al., 2001; de Almeida et al., 2013; Nunez-Parra et al., 2013; Rothermel et al., 2014; Chan et al., 2017; Cho and Linster, 2020). Cholinergic modulation within the olfactory bulb and the piriform cortex plays crucial roles in odor discrimination. Immunological lesions of HDB cholinergic neurons increase generalization between similar odors (Linster et al., 2001; Linster and Cleland, 2002). Activation of the cholinergic system is beneficial for olfactory learning (Chaudhury et al., 2009; Chapuis and Wilson, 2013; Takahashi et al., 2021), whereas pharmacological blockade of acetylcholine receptors in the olfactory bulb or the piriform cortex impairs acquisition of odor discrimination (Chaudhury et al., 2009; Chapuis and Wilson, 2013; Devore et al., 2014). In addition, the inactivation of HDB GABAergic neurons impairs habituation/dishabituation (Nunez-Parra et al., 2013). Therefore, both cholinergic and GABAergic projections are crucial for sensory processing and olfactory behaviors. However, how neural activity in HDB correlates with olfactory behaviors, especially whether and how cholinergic and GABAergic neurons are activated during different olfactory tasks remains largely unexplored.

Interestingly, previous studies have also shown that basal forebrain neurons receive projections from multiple olfactory regions and can be modulated by electrical stimulation of the olfactory bulb and cortex (Linster and Hasselmo, 2000; Zheng et al., 2018), indicating the existence of both feedforward and feedback connections between the olfactory system and the basal forebrain. *In vivo* electrophysiological recordings in awake animals have further demonstrated the dynamic nature of HDB neural activity during associative olfactory discrimination learning: basal forebrain neurons are recruited slightly before trial initiation in successful discrimination trials (Nunez-Parra et al., 2020) and baseline neural activity in the HDB increases during the acquisition phase of an odor–reward association (Devore et al., 2016). Both results indicate that basal forebrain neurons are involved in odor-associated learning. However, whether and how activity in HDB cholinergic and GABAergic neurons differs during odor-associated learning is unclear. In addition, although a recent study focusing on acetylcholine release in the HDB suggests that local cholinergic signaling is rapidly modulated during olfactory learning (Hanson et al., 2021), how cholinergic activity correlates with the stages in an olfactory task remains elusive.

In the present study, we addressed these questions by recording neural activity from cholinergic and GABAergic HDB neurons in mice undergoing passive odor exposure and odor–reward associative learning. Our data suggest that cholinergic and GABAergic neurons display distinct response dynamics during passive odor stimulation and olfactory learning, and play different roles in odor discrimination.

## Materials and Methods

### Animals

Male VGAT-Cre and C57BL/6J mice aged 10–16 weeks old were used. Mice were housed in groups and maintained on a 12 h light/dark cycle with *ad libitum* food and water except when mice were trained for the behavioral task, during which they were water restricted and allowed access to water to maintain >80% of their original weight. Mice were individually housed and allowed to recover from surgery for at least 2 weeks. All experimental procedures were carried out in accordance with protocols approved by the Xuzhou Medical University Institutional Animal Care and Use Committee.

### Virus Injection and Fiber Implant

The surgical procedures have been described in our previous studies (Case et al., 2017; Wang et al., 2019, 2020, 2022). For virus injection, mice were anesthetized with pentobarbital sodium (i.p., 90 mg/kg) and head-fixed in a stereotactic frame (RWD, Shenzhen, China). After exposure, the skull was thinned and removed carefully in the targeted brain area. The AAV virus (BrainVTA, Wuhan, China) was delivered to the HDB (AP: 0.15 mm; lateral: 1.25 mm; DV: 5.58–5.63 mm) with a syringe pump (Stoelting Quintessential Injector) connected to a 10–15 μm diameter glass micropipette, at a rate of 40 nl/min. To record or inactivate cholinergic neurons, a total volume of 300 nl of virus [AAV-ChAT-Cre and either AAV-axon-DIO-GCaMP6s/AAV-DIO-EGFP or AAV-DIO-hM4D(Gi)-mCherry/AAV-DIO-mCherry, in a 1:2 mixture] was injected into the HDB of C57BL/6J mice. To record or inactivate GABAergic neurons, 300 nl of AAV-DIO-axon-GCaMP6s/AAV-DIO-EGFP or AAV-DIO-hM4D(Gi)-mCherry/AAV-DIO-mCherry was injected into the HDB of VGAT-Cre mice. After viral delivery, the glass pipette was left in place for an additional 10 min before being slowly withdrawn. For the chemogenetic experiments, the incision was sutured after the virus injection. For fiber photometry recordings, an optical fiber [0.37 numerical aperture (NA), 200-μm diameter; Newdoon, Hangzhou, China] was implanted in the HDB after the virus injection at the same coordinates and fixed to the skull with cyanoacrylate glue and dental acrylic alongside with a custom-made aluminum headplate to allow head-fixation. Mice were then treated with lincomycin hydrochloride and lidocaine hydrochloride gel to alleviate inflammation and pain, housed individually, and allowed to recover and virus expression for at least 2 weeks. After the behavioral tests, mice were sacrificed for standard histology to confirm virus injection and fiber placement.

### Immunohistochemistry

To verify viral expression, frozen brain sections containing the HDB were prepared. The mice were anesthetized with pentobarbital sodium (i.p., 90 mg/kg) and transcardially perfused with 20 ml saline (0.9%), and then with 20 ml 4% paraformaldehyde (PFA) in PB (0.1 M, pH 7.4). After perfusion, brains were harvested, postfixed in 4% PFA for 24 h at 4°C, and then were cryoprotected with 30% sucrose in PB until the tissue sank. Brain tissue was then embedded in an OCT compound and sectioned into 30 μm slices with a vibratome (Leica Inc). Coronal brain slices containing the HDB were mounted onto microscope slides and incubated with a blocking solution (10% normal goat serum, 0.3% Triton X-100 in PBS) for 2 h at room temperature. Next, the sections were incubated with the primary antibody (goat anti-ChAT, 1:300, AB144P, Sigma-Aldrich) which was diluted in a blocking solution for 48 h at 4°C. After washout of primary antibodies, the sections were incubated with fluorescent secondary antibodies (Alexa 594-conjugated donkey anti-rabbit antibody, 1:500, A-11058, Thermo Fisher Scientific) for 2 h at room temperature. After washing, the sections were incubated with DAPI for nuclear staining, coverslipped with a 50% glycerol mounting medium, and imaged with a confocal microscope (Zeiss, LSM710).

### Fiber Photometry

The C57L/6J mice (injected with AAV-ChAT-Cre and AAV-axon-DIO-GCaMP6s/AAV-DIO-EGFP) or VGAT-Cre mice (injected with AAV-axon-DIO-GCaMP6s/AAV-DIO-EGFP) implanted with an optical fiber were head-fixed on an air-supported free-floating Styrofoam ball. The GCaMP6s signals during passive odor exposure and the behavioral tasks were monitored with a fiber photometry system (Thinkerbiotech, Nanjing, China) using methods similar to our previous studies (Wang et al., 2019, 2022). A dichroic mirror (MD498, Thorlabs) reflected a laser beam from a 488-nm laser (OBIS 488LS, Coherent) which focused through an objective lens (10× NA: 0.3; Olympus), and then coupled to an optical commutator (Doric Lenses). An input cable (200-mm o.d., NA:0.37, 1.5-m long) was connected to the implanted optical fiber and the laser power at the tip of the optical fiber was set to 50 μW. GCaMP6s fluorescence emissions were detected by a photomultiplier tube (R3896, Hamamatsu) after being bandpass filtered (MF525-39, Thorlabs). The photomultiplier tube current output was converted to a voltage by an amplifier (C7319, Hamamatsu) and was further filtered through a low-pass filter (35 Hz cutoff; Brownlee, 440). The analog voltage signals were then digitized at 500 Hz and recorded by fiber photometry software. To exclude that the cholinergic and GABAergic signals observed in mice expressing GCaMP6s were not motion artifacts, we also used a fiber photometry system with two excitation wavelengths, the calcium-dependent excitation wavelength (470 nm) and the calcium-independent isosbestic wavelength (580 nm).

### Odor Delivery

An odor delivery system (Thinkerbiotech, Nanjing, China) was used to deliver odors (Sinopharm Chemical Reagent, Shanghai, China) which were dissolved in mineral oil at 1% (v/v) dilution. A stream of charcoal-filtered air flowed over the odor which was then diluted to 1/20 by an olfactometer. The duration of odor presentation was 2 s in each trial and the inter-trial interval was 30 s. The odor presentation was synchronously controlled by the data acquisition system *via* a solenoid valve driven by a digital-to-analog converter and air was delivered to the mouse at a constant rate of 1 l/min to eliminate the effect of airflow. The temporal information of the odor delivery system was tested by a mini photo-ionization detector (PID, Aurora Scientific, Canada). The latency from the odor trigger to 10% PID maximum was around 50 ms, the rise time from 10% to 90% PID maximum was around 130 ms, and the time to return to 10% PID maximum after trigger offset (decline) was around 100 ms. Eight odors were used during passive odor exposure: isoamyl acetate, 2-heptanone, phenyl acetate, benzaldehyde, dimethyl butyric acid, n-heptane acid, n-pentanol, and 2-pentanone.

### Overview of Training and Behavioral Tasks

The animal training and behavioral tasks were carried out using methods similar to our previous studies (Wang et al., 2022; Wu et al., 2022). For fiber photometry recordings, head-fixed mice were trained on an air-supported free-floating Styrofoam ball which allowed the mice to maneuver. Mice were water restricted for 2–3 days before behavioral training (a go/go task and a go/no-go task). The body weight of the training mouse was monitored daily and maintained at 80%–85% of its initial weight. Mice performed daily sessions of the behavioral task, with each session consisting of 120 trials. Two odor pairs (isoamyl acetate vs. 2-heptanone and phenyl acetate vs. benzaldehyde) were used during the behavioral tasks. One of the two odors was pseudo-randomly delivered (maximum of two trials in a row with the same odor) on each trial which consisted of a 2-s odorant delivery period, followed by a 1-s answer period, during which the mouse could choose whether or not to lick the lick spout. Mice were first trained to lick the water spout for reward and then were trained to get water reward only during odor presentation (the go/go task). During the go/go task, the mice were rewarded by licking the water spout during the answer period when either of the odors was delivered. The water release was triggered by licking measured by a pair of infrared photobeams 0.5 s before the end of the S+ presentation. The mouse would get the water reward for 1 s and the water release would be turned off by an electromagnetic valve, ensuring that the mouse can only access water for 1 s. Next, the mice were trained to perform a go/no-go task in which they learned to discriminate the reinforced odor (S+) from the unreinforced odor (S–) to receive the water reward. In this task, mice learned to lick the water spout in go trials (S+ trials) and withhold licking in no-go trials (S– trials). Thus, in go trials, mice would get a water reward by licking (Hit), otherwise the trial would be classed as a Miss (no water reward was delivered). In no-go trials, water was never delivered regardless of whether they licked (false alarm, FA) or correctly refrained from licking during the odor (S–) presentation (correct rejection, CR). Hits and CRs were classed as correct responses and Misses and FAs were classed as wrong responses upon which a 10-s timeout punishment was introduced. Behavioral performance was evaluated in blocks of 20 trials (10 S+ and 10 S– trials presented at random), and each session included 10 blocks. The percentage correct value for each block was calculated and mice were trained to achieve a performance criterion of ≥80% correct for two consecutive blocks. The GCaMP6s signals were recorded simultaneously throughout the whole behavioral task.

For chemogenetic experiments, the behavioral tasks were performed in freely moving mice under the same training protocols. Mice were injected daily with saline or CNO (i.p., 3.3 mg/kg) 40 min before the training started. The trial was initiated by the training mouse entering the odor port and breaking a photodiode beam. Behavioral performance was evaluated in each session (day) which included 140–160 trials. During the easy task, isoamyl acetate (0.01%) and 2-heptanone (0.01%) were used as the S+ and the S–. In the difficult task, a 6:4 combination of isoamyl acetate (0.01%) and 2-heptanone (0.01%) was used as the S+ and a 4:6 combination of isoamyl acetate (0.01%) and 2-heptanone (0.01%) was used as the S–.

### Electrophysiological Recordings

#### Brain Slice Preparation

C57BL/6J and VGAT-Cre mice injected with AAVs in the HDB were used to test the responses of neurons expressing hM4D(Gi) to CNO perfusion. Mice anesthetized with pentobarbital sodium (i.p., 90 mg/kg) were subjected to cardiac perfusion with ice-cold dissection buffer saturated with 95% O_2_/5% CO_2_ containing (in mM): 85 NaCl, 2.5 KCl, 1.25 NaH_2_PO_4_, 25 NaHCO_3_, 25 glucose, 75 sucrose, 0.5 CaCl_2_, and 4 MgCl_2_. The brain was subsequently removed and slowly sliced with a vibratome (VT 1200S; Leica Inc.). Coronal brain slices containing the HDB (350 μm) were recovered at 37°C for 60 min in a chamber filled with oxygenated artificial CSF (ACSF) containing (in mM): 119 NaCl, 2.5 KCl, 1.25 NaH_2_PO_4_, 2.5 CaCl_2_, 1.3 MgCl_2_, 1.3 NaHCO_3_, and 10 glucose, equilibrated with 95% O_2_/5% CO_2_. One hour later, the holding chamber with slices was placed at room temperature and the slices were ready for patch-clamp recordings.

#### Patch-Clamp Recordings

Electrophysiological experiments were performed using the protocol described in our previous work (Wang et al., 2020, 2022). In brief, slices were transferred to a recording chamber and carbogen-saturated ACSF was perfused constantly at a flow rate of 2–3 ml/min at room temperature. The HDB in coronal brain slices was identified by visualizing the slices through a 60× water-immersion objective under near-infrared DIC illumination with an upright microscope (ECLIPSE FN1, Nikon) equipped with wide-field fluorescence to identify fluorescently labeled (Gi-mCherry) neurons. Whole-cell patch-clamp recordings were obtained with a MultiClamp 700B amplifier (Molecular Devices), a Digidata 1440 A analog-to-digital converter (Molecular Devices), and pClamp 10.4 software (Molecular Devices). Voltage traces were sampled at 10 kHz and filtered at 2 kHz. Recording electrodes had a resistance of 4–6 MΩ when filled with an intrapipette solution containing (in mM): 135 K-gluconate, 5 KCl, 0.5 CaCl_2_, 10 HEPES, 2 Mg-ATP, 0.1 GTP, and 5 EGTA, 300 mOsm, pH 7.3 adjusted with KOH. All drugs were obtained from Sigma-Aldrich. Electrophysiological data were analyzed with Clampfit 10.2 (Molecular Devices) and the “event detection feature” was used to analyze the frequency of action potentials.

### Statistical Analyses

#### Behavioral Performance

For the go/no-go task, the correct value in each block was calculated as: (number of Hit trials + number of CR trials)/total number of trials. The performance on S+ trials was calculated as number of Hit trials/(number of Hit trials + number of Miss trials). The performance on S− trials was calculated as number of CR trials/(number of FA trials + number of CR trials).

#### Analysis of Fiber Photometry Data

Data were exported as MATLAB .mat files and segmented according to the onset of odor stimulation on individual trials. We derived the values of fluorescence change (ΔF/F) by calculating (F-F_0_)/F_0_, where F_0_ is the baseline fluorescence signal averaged over a 5-s-long control time window, which preceded the onset of odor stimulation. ΔF/Fs are presented as heat maps or trial-averaged traces. Averaged ΔF/F value for 4 s from the onset of odor deliverywas quantified as the area under the peak (the area under the curve, AUC).

#### ROC Analysis

We used receiver operating characteristic (ROC) analysis to assess the classification of the responses evoked by two odors within an odor pair (Gadziola et al., 2015; Wang et al., 2019). ROCs were estimated using the ROC function from the MATLAB exchange. The area under the ROC (auROC) is a nonparametric measure of the discriminability of two distributions. The area under the ROC curve was defined as ranging from 0.5 to 1.0. A value of 0.5 indicates completely overlapping distributions, whereas a value of 1.0 indicates perfect discriminability.

#### Calculation of Differences in ΔF/F

We used the difference in ΔF/F to assess the extent of the divergence in the responses during the go/no-go and go/go tasks. In the go/no-go task, the responses in S+ trials and S– trials were defined as Res S+ and Res S–, respectively. The difference in ΔF/F was calculated as follows: ABS (Res S+ − Res S–)/[ABS (Res S+) + ABS (Res S–)], where ABS represents the absolute value. Similarly, in the go/go task, the responses in odor A trials and odor B trials were defined as Res A and Res B, respectively. The difference in ΔF/F was calculated as follows: ABS (Res A − Res B)/[ABS (Res A) + ABS (Res B)].

#### Statistical Tests

All statistical analyses were performed in MATLAB. The Shapiro-Wilk test was used to assess the normality of the data. We used two-way ANOVA, Wilcoxon signed-rank test, and paired *t*-test; all tests were two-sided. All data in the study are presented as the mean ± SEM.

## Results

### Both Cholinergic and GABAergic Responses Decrease Over Time With Repeated Odor Exposure

**Figure 1:**
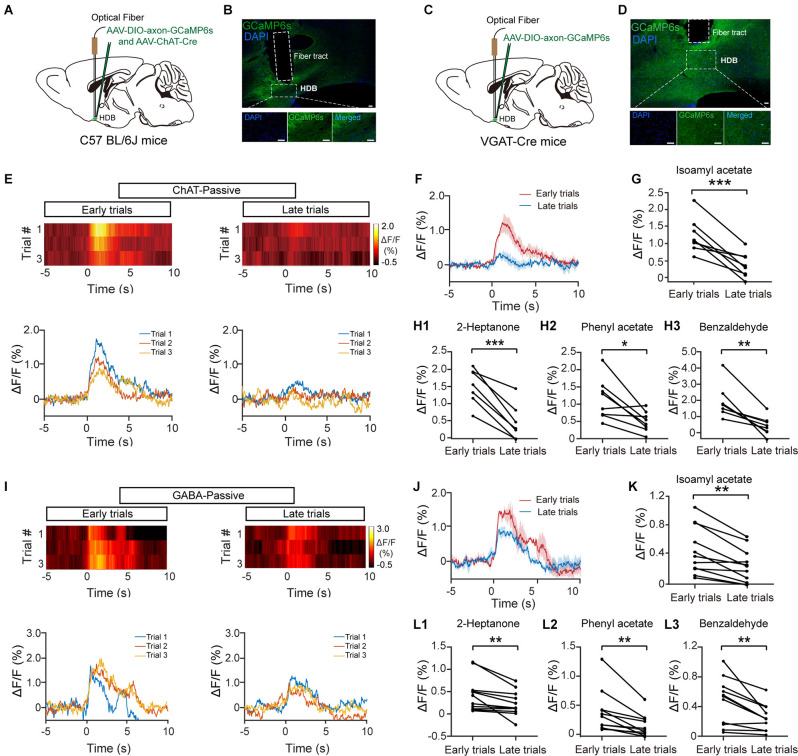
The response profiles of HDB cholinergic and GABAergic neurons during passive exposure. **(A)** Diagram of virus injection. To record the calcium responses of HDB cholinergic neurons, AAV-ChAT-Cre and AAV-DIO-axon-GCaMP6s were injected into the HDB of C57BL/6J mice. **(B)** Sections showing expression of GCaMP6s in the HDB from a C57BL/6J mouse following virus injection. Scale bar: 50 μm. **(C)** Diagram of virus injection. To record the calcium responses of HDB GABAergic neurons, AAV-DIO-axon-GCaMP6s were injected into the HDB of VGAT-Cre mice. **(D)** Sections showing expression of GCaMP6s in the HDB from a VGAT-Cre mouse following virus injection. Scale bar: 50 μm. **(E,I)** Heat maps and traces of ΔF/F for the first three trials (early trials) and the last three trials (late trials) in cholinergic neurons **(E)** or GABAergic neurons **(I)** from representative mice under repeated odor (Isoamyl acetate) exposure. **(F,J)** The trial-averaged traces of ΔF/F in **(E)** and **(I)**, respectively. **(G,K)** The odor (Isoamyl acetate) responses in cholinergic **(G)** and GABAergic **(K)** neurons decreased in late trials compared with early trials [**(G)** paired *t*-test, *t*_(7)_ = 5.53, *P* = 8.9 × 10^−4^; **(K)** paired *t*-test, *t*_(10)_ = 4.59, *P* = 0.001]. **(H,L)** The ΔF/F in cholinergic **(H)** and GABAergic **(L)** neurons also decreased upon repeated exposure to other odors (2-Heptanone, Phenyl acetate, and Benzaldehyde, respectively) [**(H_1_)** paired *t*-test, *t*_(7)_ = 8.37, *P* = 6.8 × 10^−5^; **(H_2_)** paired *t*-test, *t*_(7)_ = 3.37, *P* = 0.012; **(H_3_)** paired *t*-test, *t*_(7)_ = 4.68, *P* = 0.0023; **(L_1_)** Wilcoxon signed-rank test, *z* = 2.85, *P* = 0.0044; **(L_2_)** Wilcoxon signed-rank test, *z* = 2.93, *P* = 0.0033; **(L_3_)** paired *t*-test, *t*_(10)_ = 3.60, *P* = 0.0049]. *P < 0.05, **P < 0.01, ***P < 0.001.

**Figure 2:**
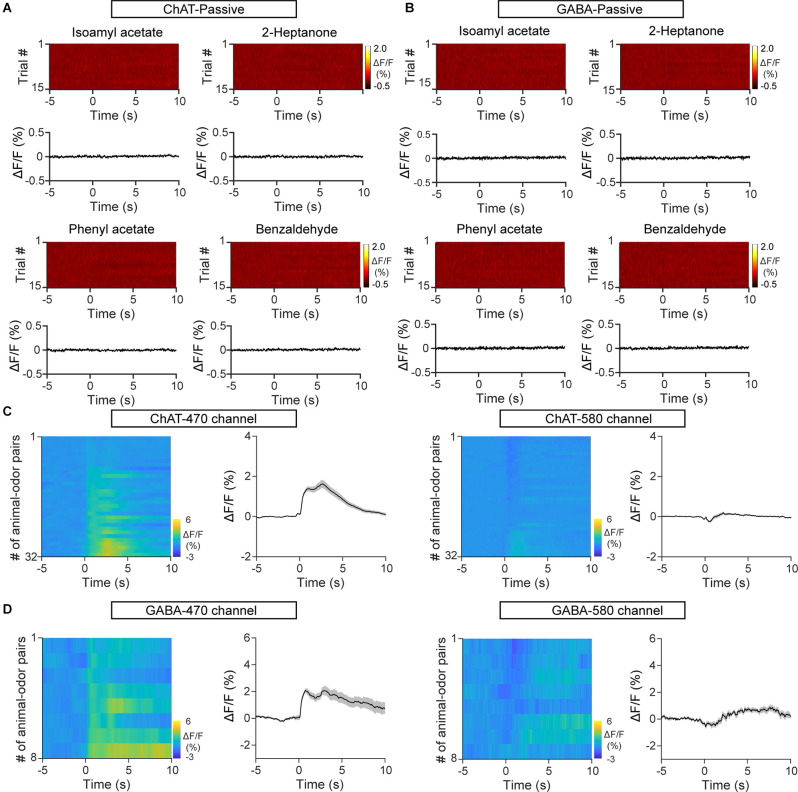
Control mice expressing EGFP and the second fluorescent channel show no changes influorescence upon odor exposure. **(A)** Heat maps andtrial-averaged traces of ΔF/F from a representative C57BL/6J mouse injected with AAV-ChAT-Cre and AAV-DIO-EGFP. **(B)** Heat maps and trial-averaged traces of ΔF/F from a representative VGAT-Cre mouse injected with AAV-DIO-EGFP. **(C)** Heat maps and trial-averaged traces of ΔF/F recorded from C57BL/6J mice injected with AAV-ChAT-Cre and AAV-DIO-axon-GCaMP6s using the targeted recording channel (470 nm) and a second fluorescent channel (580 nm). *n* = 32 animal–odor pairs from four mice. **(D)** Heat maps and trial-averaged traces of ΔF/F recorded from a VGAT-Cre mouse injected with AAV-DIO-axon-GCaMP6s using the targeted recording channel and the second fluorescent channel. *n* = 8 animal–odor pairs from one mouse.

GCaMP6s signals of cholinergic/GABAergic HDB neurons were monitored by fiber photometry in awake, head-fixed mice. GCaMP6s expression was genetically restricted to cholinergic neurons by injecting a composite virus solution (AAV-ChAT-Cre and AAV-DIO-axon-GCaMP6s) into the HDB in C57BL/6J mice (Figures 1A,B). Similarly, VGAT-Cre mice injected with AAV-DIO-axon-GCaMP6s were used to record Ca^2+^ signals in HDB GABAergic neurons (Figures 1C,D). First, we characterized the population response of cholinergic/GABAergic neurons in awake, head-fixed mice during passive odor exposure. The animals were exposed to a 2-s pulse of odor stimulation for 120 successive trials. Both cholinergic/GABAergic neurons displayed a rapid increase in the Ca^2+^ signal upon odor delivery. However, the responses decreased as the trials progressed (Figures 1E,F,I,J). We compared the averaged ΔF/F for the first three trials with that for the last three and found that the ΔF/F was significantly lower at the end of the experiment (Figures 1G,K; G: paired *t*-test, *P* = 8.9 × 10^−4^; K: paired *t*-test, *P* = 0.001). Decreased responses were also observed for other odors (Figures 1H,L; H_1_: paired *t*-test, *P* = 6.8 × 10^−5^; H_2_: paired *t*-test, *P* = 0.012; H_3_: paired *t*-test, *P* = 0.0023; L_1_: Wilcoxon signed-rank test, *P* = 0.0044; L_2_: Wilcoxon signed-rank test, *P* = 0.0033; L_3_: paired *t*-test, *P* = 0.0049). Similar results were obtained when we compared the averaged ΔF/F for the first five trials with that for the last five trials (Supplementary Figures 1A,B; A: Isoamyl acetate, paired *t*-test, *P* = 0.0019; 2-Heptanone, paired *t*-test, *P* = 7.57 × 10^–5^. B: Isoamyl acetate, paired *t*-test, *P* = 0.0010; 2-Heptanone, paired *t*-test, *P* = 0.0076). We used mice that expressed EGFP in HDB cholinergic and GABAergic neurons as a control, which did not show any changes in fluorescence during passive odor exposure (Figures 2A,B). In addition, we also used a second fluorescent channel (580 nm) as a control. We found that while odor evoked strong responses in the targeted recording channel, almost no responses were found in the control channel (Figures 2C,D). These results suggest that the cholinergic and GABAergic signals observed in mice expressing GCaMP6s were not motion artifacts. All these results suggest that cholinergic and GABAergic HDB neurons display decreased responses to odors during repeated passive exposure.

### Responses of Cholinergic Neurons Remain Stable During Olfactory Associative Learning

**Figure 3:**
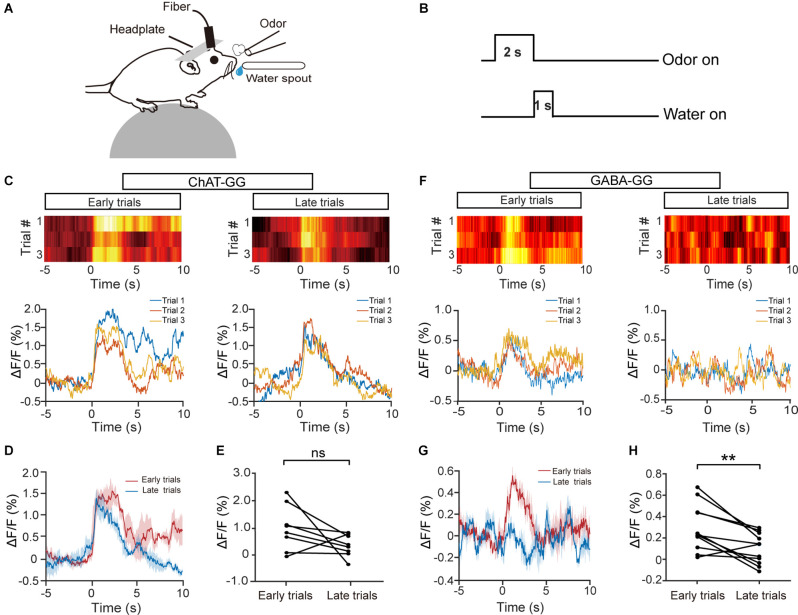
The response profiles of HDB cholinergic and GABAergic neurons during the go/go task. **(A)** Diagram of the experimental paradigm. **(B)** Timeline for a single trial in the go/go task. **(C,F)** Representative heat maps and corresponding traces of the cholinergic **(C)** and GABAergic **(F)** responses in the first three trials (early trials) and the last three trials (late trials) of the go/go task. **(D,G)** The trial-averaged traces of ΔF/F in **(C)** and **(F)**, respectively. **(E,H)** ΔF/F in cholinergic **(D)** or GABAergic **(G)** neurons across all animal–odor pairs in the early trials and the late trials [**(E)** Paired *t*-test, *t*_(7)_ = 1.80, *P* = 0.11; **(H)** paired *t*-test, *t*_(10)_ = 3.69, *P* = 0.0042]. ns, no significance, **P < 0.01.

Previous studies have shown that cholinergic neurons are recruited during associative learning (Devore et al., 2016; Nunez-Parra et al., 2020). Thus, we next examined the response profiles of HDB cholinergic neurons during olfactory associative learning (Figure 3A). Mice were trained to learn a go/go task in which they could receive the water reward by licking the water spout within a defined time window after odor presentation (Figure 3B). We recorded Ca^2+^ signals from cholinergic/GABAergic neurons during a 120-trial session in animals proficient in the go/go task and compared the average ΔF/F from the first three trials with that from the last three trials (Figures 3C,F). Interestingly, while GABAergic neurons showed decreased responses in the late stage of the session (Figures 3G,H; H: paired *t*-test, *P* = 0.0042), the responses of cholinergic neurons were relatively stable across the session (Figures 3D,E; E: paired *t*-test, *P* = 0.11). Similar results were obtained when we compared the averaged ΔF/F for the first 10 trials with that for the last 10 trials (Supplementary Figures 1C,D; C: Isoamyl acetate, paired* t*-test, *P* = 0.14; 2-Heptanone, paired *t*-test, *P* = 0.51. D: Isoamyl acetate, paired *t*-test, *P* = 0.0066; 2-Heptanone, paired *t*-test, *P* = 0.012). These results indicate that the responses of HDB cholinergic neurons may reflect attention to rewarded odors during olfactory associative learning.

The different odor response properties between cholinergic and GABAergic neurons might be due to the difference in bleaching of the marker protein. To exclude this possibility, we analyzed the baseline Ca^2+^ fluorescence change during a session (120 trials). We found that both cholinergic and GABAergic fluorescence decreased slightly, which might be resulted from the bleaching of the marker protein. However, there was no significant difference between the baseline cholinergic and GABAergic fluorescence (Supplementary Figure 2A). Further analysis indicated that both the cholinergic and GABAergic fluorescence was negatively correlated with the trial numbers, whereas there was no significant difference between the two groups (Supplementary Figure 2B; Wilcoxon signed-rank test, *P* = 0.31). Thus, the different response properties between cholinergic and GABAergic neurons in the go/go task were not due to the difference in bleaching.

### Cholinergic Neurons Develop Separated Responses to the S+ and S− During Odor Discrimination Learning

**Figure 4:**
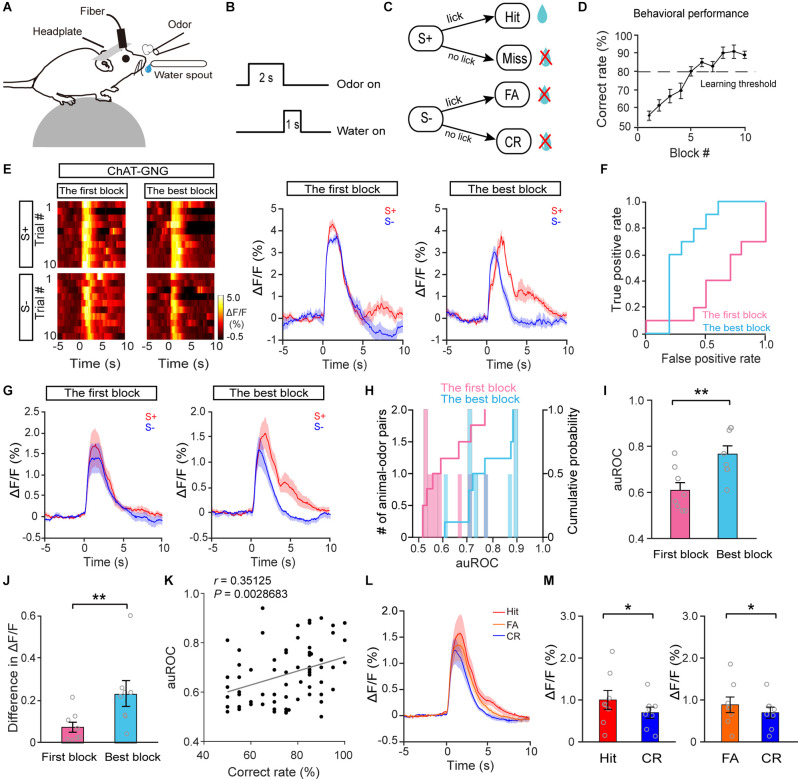
The response profiles of HDB cholinergic neurons in S+ trials and S–trials during the go/no-go task. **(A)** Diagram of the experimental paradigm. **(B)** Timeline for a single trial inthe go/no-go task. **(C)** Schematic of the behavioral paradigm. If an S+ was presented and the mouse responded with licking, a water reward was delivered. If an S– was presented, no water reward was delivered regardless of the mouse’s actions. **(D)** Odor discrimination performance during the last session in the go/no-go task. The mean percentage correct rates are plotted for each block of 20 trials. The learning threshold is indicated by a dashed line. **(E)** Representative heat maps and corresponding traces of cholinergic responses in S+ and S– trials in the first block and in the best block during the go/no-go task. **(F)** ROC graph for S+ and S–responses in the first block and the best block shown in **(E)**. **(G)** Averaged traces of cholinergic responses in S+ and S– trials in the first block and the best block across all animal–odor pairs (*n* = 16 animal–odor pairs from eight mice). **(H)** Histograms and cumulative probability of auROCs in the first block and the best block. **(I,J)** Comparison of auROCs **(I)** and difference in ΔF/F **(J)** in the first block and the best block [**(I)** Paired *t*-test, *t*_(7)_ = −3.81, *P* = 0.0067; **(J)** paired *t*-test, *t*_(7)_ = −3.54, *P* = 0.0095]. **(K)** The auROCs of cholinergic responses plotted against the correct rate during the go/no-go task (Linear regression, *r* = 0.35, *P* = 0.0029, *n* = 70 blocks). **(L,M)** Comparison of averaged cholinergic responses in Hit, FA, and CR trials [**(M)** Hit vs. CR, Wilcoxon signed-rank test, *z* = 2.52, *P* = 0.012, FA vs. CR, paired *t*-test, *t*_(7)_ = −2.54, *P* = 0.038]. *P < 0.05, **P < 0.01.

Our data suggest that the responses of cholinergic neurons to odor presentation decrease with repeated passive odor exposure but remain stable when attention is required to obtain a reward, raising the question of whether cholinergic neurons might respond differently to rewarded trials vs. unrewarded trials during an odor-discrimination task. To address this question, we recorded the responses of HDB cholinergic neurons while head-restrained mice learned to respond differently to odors in a go/no-go odor discrimination task (Figure 4A; see also “Materials and Methods” Section). Thirsty mice were trained to lick the water spout for reward upon a rewarded odor (S+) presentation and refrain from licking upon an unrewarded odor presentation (Figure 4B). The possible behavioral responses were Hit, correct rejection (CR), false alarm (FA), and miss (Figure 4C). The behavioral performance was assessed by calculating the percentage of correct responses to the S+ and S− odors in blocks of 20 trials in which 10 S+ and 10 S− odors were delivered randomly. The learning curve across all trained mice is shown in Figure 4D. The behavioral performance of mice improved from near chance levels (50% correct) in Block 1 to well above the learning threshold (80% correct) in Block 10.

Figure 4E shows the responses of cholinergic neurons for a representative mouse learning to discriminate between the S+ and S−. The traces were sorted into trials where the mouse was beginning to differentiate the odors (left, the first block) and trials where the mouse was proficient in discriminating the odors (right, the block with the highest correct rate, the best block). We found that the neural responses to S+ trials and S− trials were similar during the first block. However, the responses diverged once the mouse had learned the go/no-go task (Figure 4E). To compare the classification of the responses during S+ trials and S− trials, we performed an ROC analysis. For the example shown in Figure 4E, the auROC, representing the difference in responses to S+ trials and S− trials, was larger in the best block than in the first block (Figure 4F, the first block: 0.56, the best block: 0.88), indicating that discriminability of the responses in S+ trials and S− trials was improved after the mouse learned the go/no-go task. Increased auROCs after mice learned the task were observed across all animals (Figures 4G–I; I: paired *t*-test, *P* = 0.0067, *n* = 16 animal–odor pairs from eight mice). To quantify the extent of the response difference between S+ trials and S− trials, we calculated the difference in averaged ΔF/F between 0 and 4 s. Consistent with the results from the auROC analysis, the difference in averaged ΔF/F was larger in the best block than in the first block (Figure 4J, paired *t*-test, *P* = 0.0095). Both the results of auROC analysis and the difference in averaged ΔF/F demonstrated that the responses in S+ trials and S− trials became separated once mice were proficient in the go/no-go task. We then examined whether there was a correlation between the correct rates (in each block) and auROCs of cholinergic responses during the go/no-go task. The results showed that the auROCs were positively correlated with learning accuracy (Figure 4K; linear regression, *r* = 0.35, *P* = 0.0029), indicating that cholinergic responses in S+ trials and S− trials tend to diverge with higher learning accuracy. The similarity of the cholinergic response in S+ trials and S− trials at the beginning of the go/no-go task may be because the animals pay attention during both types of trials when they have not yet learned to discriminate between the S+ and S−. Once the mice have learned the go/no-go task, they may pay more attention to S+ trials than to S− trials, presenting divergent responses to S+ trials and S− trials.

In the go/no-go task, the behavioral responses on most of the trials were Hit, CR, or FA, with very few Miss trials (12/1,394, 0.86%). When we compared cholinergic responses in Hit, CR, and FA trials across the session (Figure 4L), we found that the responses in Hit and FA trials were significantly larger than the responses in CR trials (Figure 4M; Hit vs. CR: Wilcoxon signed-rank test, *P* = 0.012; FA vs. CR: paired *t*-test, *P* = 0.038). The smaller cholinergic responses in CR trials may be because the animals have learned to ignore the S− (CR trials) and pay attention to the S+ (Hit trials); before the mice have not learned the task, they presumably pay attention to both the S− (FA trials) and the S+ (Hit trials). Therefore, these data suggest that responses in HDB cholinergic neurons tend to be larger in trials during which the animals need to pay attention.

In the go/go and go/no-go tasks, odor delivery was usually followed by licking, this raises the possibility that the odor responses may be caused or affected by licking during the odor stimulation. We thus analyzed the lick signals and fluorescence signals in the go/go and go/no-go tasks. In the go/go task, we found that fluorescence signals were always earlier than lick signals (Supplementary Figures 3A–C), indicating that fluorescence signals can be independent of licking signals. Importantly, in the go/no-go task, there were almost no lick signals in CR trials, and there were very strong fluorescence responses (Supplementary Figure 3D). Also, for both Hit and FA trials, the fluorescent signals increased before the lick onset, and the latency from odor onset to 50% peak of the ΔF/F in odor-locked was significantly different from that in lick-locked (Supplementary Figures 3B,C,E,F; B: paired *t*-test, *P* = 0.0012; C: Wilcoxon signed-rank test, *P* = 0.0012; E: paired *t*-test, *P* = 1.51 × 10^−6^; F: paired *t*-test, *P* = 2.21 × 10^−4^). All these results suggest that the odor response recorded by fiber photometry in the present study is independent of licking and the separated cholinergic responses to S+ trials and S− trials are not contributed by licking.

**Figure 5:**
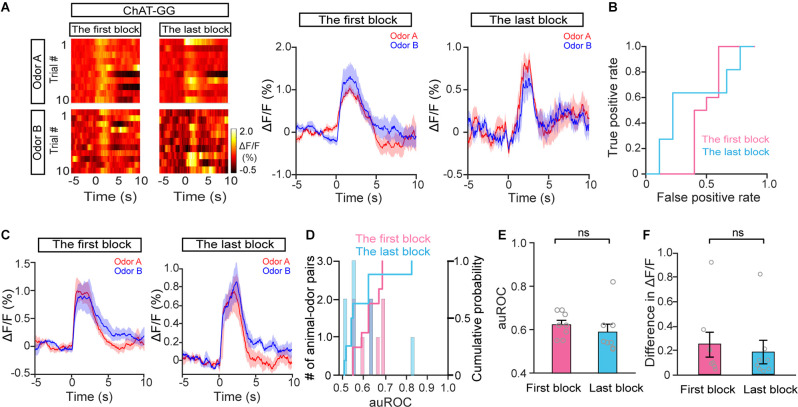
The response profiles of HDB cholinergic neurons in Odor A trials and Odor B trials during the go/go task. **(A)** Representative heat maps and corresponding traces of cholinergic responses in Odor A and Odor B trials in the first block and the last block during the go/go task. **(B)** ROC graph for the Odor A and Odor B responses in the first block and the last block shown in **(A)**. **(C)** Averaged traces of cholinergic responses in Odor A and Odor B trials in the first block and the last block across all animal–odor pairs (*n* = 16 animal–odor pairs from eight mice). **(D)** Histograms and cumulative probability of auROCs in the first block and the last block. **(E** and **F)** Comparison of auROCs **(E)** and difference in ΔF/F **(F)** in the first block and the last block [**(E)** Wilcoxon signed-rank test, *z* = 1.40, *P* = 0.16 **(F)** paired *t*-test, *t*_(7)_ = 0.53, *P* = 0.61]. ns, no significance.

In the go/no-go task, we compared cholinergic responses in the first block and in the best block; however, the animals were in different behavioral states, e.g., thirst, during these two periods. To exclude the possibility that the responses to S+ trials and S− trials differed between the first block and the best block because of general behavioral state differences, we compared cholinergic responses in the first block and the last block during the go/go task, in which animals also received water and became satiated. The responses in the odor A and odor B trials were similar during both the first block and the last block in a representative mouse (Figures 5A,B; auROC: the first block, 0.41; the last block, 0.52). Further analysis indicated that neither the auROC values nor the difference in ΔF/F across all animal–odor pairs were significantly different between the first block and the last block during the go/go task (Figures 5C–F; E: Wilcoxon signed-rank test, *P* = 0.16; F: paired *t*-test, *P* = 0.61, *n* = 16 animal–odor pairs from eight mice), indicating no separated responses were found in the go/go task. Therefore, the divergent responses of S+ and S− trials in HDB cholinergic neurons likely reflect changes in attention across the session, as opposed to changes in behavioral states such as thirst.

### GABAergic Neurons Have Similar Responses to the S+ and S− During Odor Discrimination Learning

**Figure 6:**
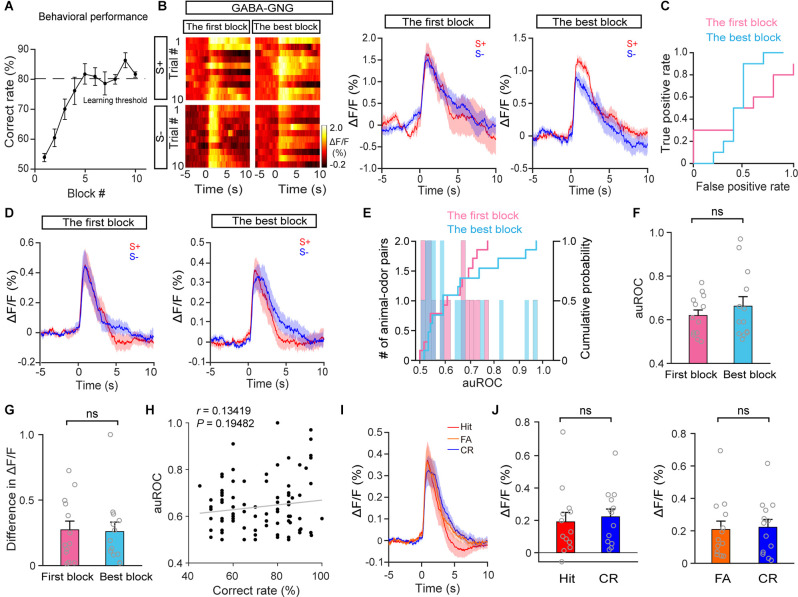
The response profiles of HDB GABAergic neurons in S+ trials and S– trials during the go/no-go task. **(A)** Odor discrimination performance during the last session in the go/no-go task. **(B)** Representative heat maps and corresponding traces of GABAergic responses in S+ and S–trials in the first block and the best block during the go/no-go task. **(C)** ROC graph for the S+ and S– responses in the first block and the best block shown in **(B)**. **(D)** Averaged traces of GABAergic responses in S+ and S– trials in the first block and the best block across all animal–odor pairs (*n* = 26 animal–odor pairs from 13 mice). **(E)** Histograms and cumulative probability of auROCs in the first block and the best block. **(F,G)** Comparison of auROCs **(F)** and difference in ΔF/F **(G)** in the first block and the best block [**(F)** Paired *t*-test, *t*_(12)_ = −0.78, *P* = 0.45; **(G)** paired *t*-test, *t*_(12)_ = 0.12, *P* = 0.91]. **(H)** The auROCs of GABAergic responses plotted against the correct rate during the go/no-go task (Linear regression, *r* = 0.13, *P* = 0.19, *n* = 95 blocks). **(I,J)** Comparison of averaged cholinergic responses in Hit, FA, and CR trials [**(J)** Hit vs. CR, paired *t*-test, *t*_(12)_ = −0.80, *P* = 0.44, FA vs. CR, Wilcoxon signed-rank test, *z* = −0.035, *P* = 0.97]. ns, no significance.

Next, we examined the responses of HDB GABAergic neurons during the go/no-go task. Figure 6A shows that all mice learned to discriminate odors successfully. The response of GABAergic neurons in S+ trials and S− trials was similar both during the first block and during the best block in a representative mouse (Figures 6B,C; auROC: the first block, 0.5; the best block, 0.46). Further analysis indicated that neither the auROC value nor the difference in ΔF/F across all animal–odor pairs was significantly different between the first block and the best block during the go/no-go task (Figures 6D–G; F: paired *t*-test, *P* = 0.13; G: paired *t*-test, *P* = 0.91, *n* = 26 animal–odor pairs from 13 mice), indicating that the separated responses in S+ trials and S− trials were not found in HDB GABAergic neurons. In addition, no correlation between accuracy and auROCs was observed in GABAergic responses (Figure 6H; linear regression, *r* = 0.13, *P* = 0.19). We also compared GABAergic responses in Hit, CR, and FA trials across the session (Figure 6I) and found that the responses in Hit and FA trials were not significantly different from that in CR trials (Figure 6J; Hit vs. CR: paired *t*-test, *P* = 0.44; FA vs. CR: Wilcoxon signed-rank test, *P* = 0.97). Therefore, these data indicate that, unlike the responses of cholinergic neurons, the responses of GABAergic neurons to the S+ and S− do not separate after mice have learned the odor-discrimination task.

We also analyzed the lick signals and GABAergic fluorescence signals in the go/go and go/no-go tasks (Supplementary Figure 4), and the results indicate that the odor-evoked GABAergic response recorded by fiber photometry is independent of licking.

### Inactivation of HDB Cholinergic Neurons Impairs Odor Discrimination

**Figure 7:**
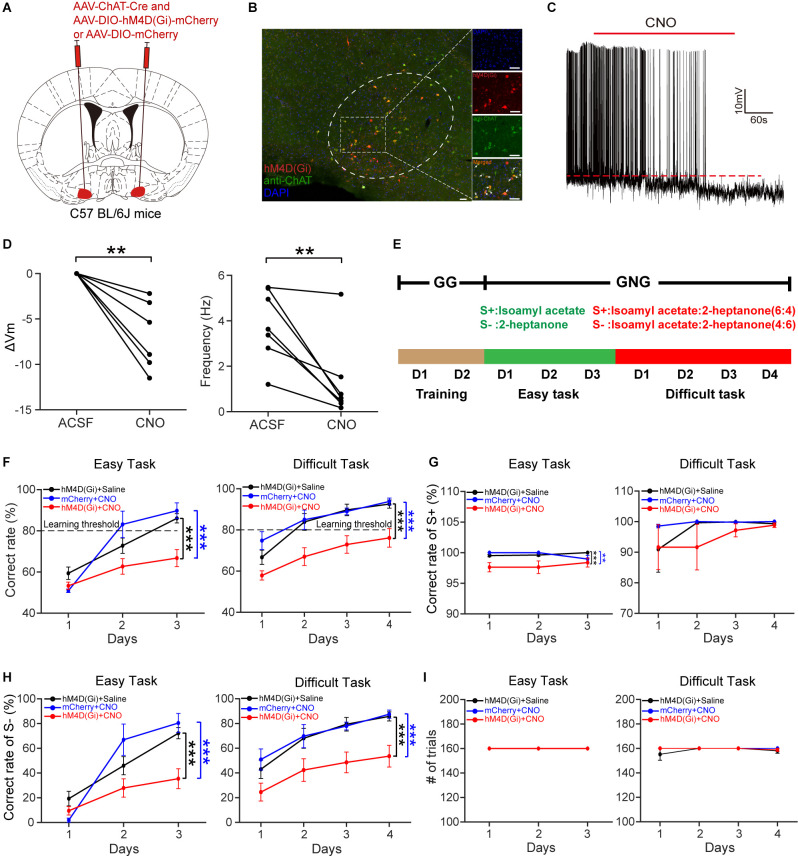
Chemogenetic inactivation of HDB cholinergic neurons impairs odor discrimination. **(A)** Schematic of virus injection. AAV-ChAT-Cre and AAV-DIO-hM4D (Gi)-mCherry/AAV-DIO-mCherry were injected into bilateral HDB of C57BL/6J mice. **(B)** Expression of hM4D(Gi)-mCherry in HDB cholinergic neurons. The white arrows indicate merged neurons. Scale bar:50 μm. **(C)** A representative trace recorded from acurrent-clamped neuron expressing hM4D(Gi). **(D)** Comparison of the change in membrane potential (ΔVm) and frequency of action potentials before and after the application of CNO (Left:paired *t*-test, *t*_(6)_ = 5.32,*P* = 0.0018; Right: paired* t*-test,*t*_(6)_ = 3.93, *P* = 0.0077;*n* = 7 cells from four mice). **(E)** Schematic of theolfactory discrimination task. **(F)** Comparison of odordiscrimination performance between control (black, *n* = 13 mice; blue, *n* = 6 mice) and experimental (red, *n* = 13 mice) mice during the easy task (left) and the difficult task (right) (Left: two-way ANOVA, *F*_(2, 91)_ = 12.3, *P* = 1.88 × 10^−5^; Right: *F*_(2, 122)_ = 25.9, *P* = 4.16 × 10^−10^). **(G)** Comparison of correct rates in S+ trials between control and experimental mice during the easy task and the difficult task (two-way ANOVA, Easy task: *F*_(2, 91)_ = 9.39, *P* = 2 × 10^−4^, Difficult task: *F*_(2, 122)_ = 0.93, *P* = 0.40). **(H)** Comparison of correct rates in S– trials between the three groups of mice (two-way ANOVA, Easy task: *F*_(2, 91)_ = 10.68, *P* = 6.84 × 10^−5^; Difficult task: *F*_(2, 122)_ = 18.94, *P* = 6.9 × 10^−8^). **(I)** Comparison of the number of trials completed by the animals in the three groups during the go/no-go task (two-way ANOVA, Difficult task: *F*_(2, 122)_ = 0.93, *P* = 0.40). **P < 0.01, ***P < 0.001.

The above results suggest that HDB cholinergic neurons may play an important role in odor discrimination. This prompted us to explore the influence of cholinergic neuron dysfunction on odor discrimination. We injected a composite virus solution [AAV-ChAT-Cre and AAV2/9-DIO-hM4D(Gi)-mCherry/AAV2/9-DIO-mCherry] into the bilateral HDB of C57BL/6J mice (Figure 7A). Four weeks after virus injection, hM4D(Gi)-mCherry expression was observed in the HDB (Figure 7B). Then we tested the effect of clozapine N-oxide (CNO) on the activity of cholinergic neurons expressing hM4D(Gi) *in vitro* (Figure 7C). Perfusion of CNO (10 μM) significantly decreased the membrane potential and the frequency of action potentials (Figure 7D; ΔVm: paired *t*-test, *P* = 0.0018; Frequency: paired *t*-test, *P* = 0.0077). To explore the influence of cholinergic neurons’ inactivation on odor discrimination, we conducted an odor discrimination assay that lasted for 9 days, including the training period (2 days), the easy task (3 days), and the difficult task (4 days) in freely moving mice. As in our previous study (Sun et al., 2019; Wang et al., 2022; Wu et al., 2022), mice were first trained to undergo a go/go task, then the mice were trained to perform a go/no-go task, including the easy task (S+: isoamyl acetate; S−: 2-heptanone) and a difficult task (S+: isoamyl acetate: 2-heptanone = 6:4; S−: 2-heptanone: isoamyl acetate = 6:4; Figure 7E). The percent correct rate during the go/no-go task was compared between experimental (Gi + CNO) and control (Gi + saline, mCherry + CNO) animals. Mice were injected daily with saline or CNO (i.p., 3.3 mg/kg) 40 min before the training started. We found that the experimental mice could not learn to discriminate between the S+ and S−, even in the easy task: the percent correct rate was significantly lower in experimental mice than in control mice both in the easy task and in the difficult task (Figure 7F; easy task: two-way ANOVA, Gi + saline vs. Gi + CNO, *P* < 1 × 10^−4^, mCherry + CNO vs. Gi + CNO, *P* =1 × 10^−4^, Gi + saline vs. mCherry + CNO, *P* = 0.81; difficult task: two-way ANOVA, Gi + saline vs. Gi + CNO, *P* < 1 × 10^−4^, mCherry + CNO vs. Gi + CNO, *P* < 1 × 10^−4^, Gi + saline vs. mCherry + CNO, *P* = 0.83). Further analysis indicated that while reductions in correct responses in both S+ and S− trials were responsible for the impaired odor discrimination in experimental mice during the easy task, impaired performance during the difficult task was caused by a reduction in correct responses in S− trials only (Figures 7G,H; G: Easy task: two-way ANOVA, Gi + saline vs. Gi + CNO, *P* = 4 × 10^−4^, mCherry + CNO vs. Gi + CNO, *P* = 0.0082, Gi + saline vs. mCherry + CNO, *P* = 0.99; Difficult task: two-way ANOVA, Gi + saline vs. Gi + CNO, *P* = 0.65, mCherry + CNO vs. Gi + CNO, *P* = 0.41, Gi + saline vs. mCherry + CNO, *P* = 0.83; H: Easy task: two-way ANOVA, Gi + saline vs. Gi + CNO, *P* = 2 × 10^−4^, mCherry + CNO vs. Gi + CNO, *P* = 4 × 10^−4^, Gi + saline vs. mCherry + CNO, *P* = 0.81; Difficult task: two-way ANOVA, Gi + saline vs. Gi + CNO, *P* < 1 × 10^−4^, mCherry + CNO vs. Gi + CNO, *P* < 1 × 10^−4^, Gi + saline vs. mCherry + CNO, *P* = 0.92). The differences in percent correct rates were not due to distinct training intensity because the numbers of training trials between experimental and control mice were not significantly different (Figure 7I, Difficult task: two-way ANOVA, *P* = 0.40). These results indicate that the inactivation of HDB cholinergic neurons impairs odor discrimination.

### Inactivation of HDB GABAergic Neurons Has No Effect on Odor Discrimination

**Figure 8:**
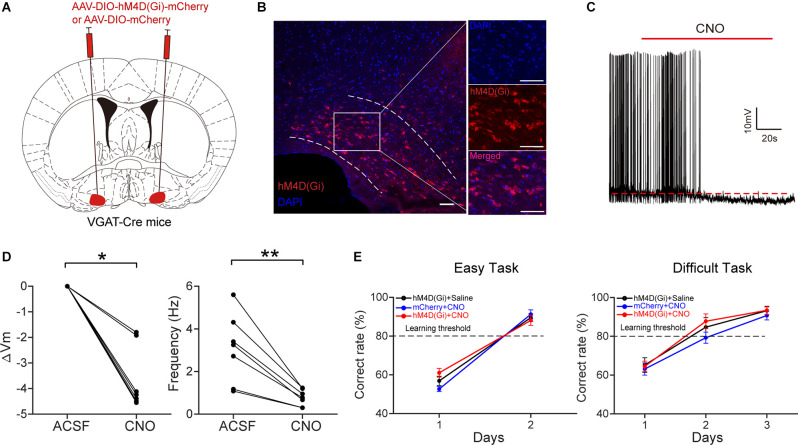
Chemogenetic inactivation of HDB GABAergic neurons has no effect on odor discrimination. **(A)** Schematic of virus injection.AAV-DIO-hM4D(Gi)-mCherry/AAV-DIO-mCherry was injected into bilateral HDB of VGAT-Cre mice. **(B)** Expression ofhM4D(Gi)-mCherry in HDB GABAergic neurons. Scale bar: 50 μm.**(C)** A representative trace recorded from a current-clamped neuron expressing hM4D(Gi). **(D)** Comparison of the change in membrane potential (ΔV_m_) and frequency of action potentials before and after the application of CNO (Left: Wilcoxon’s sign rank test, *z* = 2.366, *P* = 0.018; Right: paired* t*-test, *t*_(6)_ = 4.8145, *P* = 0.003, *n* = 7 cells from four mice). **(E)** Comparison of odor discrimination performance between control (black, *n* = 8 mice; blue, *n* = 6 mice) and experimental (red, *n* = 9 mice) mice during the easy task (left) and the difficult task (right) (two-way ANOVA, Easy task: *F*_(2, 44)_ = 0.62, *P* = 0.54; Difficult task:*F*_(2, 67)_ = 1.39, *P* = 0.26). *P < 0.05, **P < 0.01.

We next examined whether the inactivation of HDB GABAergic neurons affects odor discrimination behavior. We injected AAV2/9-DIO-hM4D(Gi)-mCherry/AAV2/9-DIO-mCherry into the bilateral HDB of VGAT-Cre mice (Figure 8A). Four weeks after virus injection, hM4D(Gi)-mCherry expression was observed in the HDB (Figure 8B). Then we tested the effect of CNO on the activity of GABAergic neurons expressing hM4D(Gi) *in vitro* (Figure 8C). Perfusion of CNO (10 μM) significantly decreased the membrane potential and the frequency of action-potentials (Figure 8D; ΔVm: Wilcoxon’s sign rank test, *P* = 0.018; Frequency: paired* t*-test, *P* = 0.003). We trained the mice on the go/no-go task and found that the percent correct rates were not significantly different between the experimental mice and the control mice (Figure 8E; easy task: two-way ANOVA, *P* = 0.54; difficult task: two-way ANOVA, *P* = 0.26). These results indicate that, unlike inactivation of HDB cholinergic neurons, inactivation of HDB GABAergic neurons had no effect on odor discrimination behavior.

## Discussion

Both cholinergic and GABAergic neurons in the HDB send long-distance projections to modulate the neural activity and function of other brain areas (Do et al., 2016; Villar et al., 2021; De Saint Jan, 2022). Although the modulatory effects of these two projections on the olfactory system have been intensively studied (Fletcher and Chen, 2010; Ma and Luo, 2012; Nunez-Parra et al., 2013), a comparison of the odor responses and function of these two projections during different behavioral states is still lacking. Here we used fiber photometry to demonstrate that HDB cholinergic and GABAergic neurons display distinct response dynamics during odor discrimination learning. While both cholinergic and GABAergic responses decreased over time upon repeated passive odor exposure, they displayed different patterns during odor-association learning: during a go/go task, responses in cholinergic neurons remained stable whereas responses in GABAergic neurons decreased as the session progressed, as they did during passive exposure. Moreover, during a go/no-go odor discrimination task, the responses of cholinergic neurons to the S+ and S− separated once the mice learned to discriminate the two odors correctly. Chemogenetic inactivation of HDB cholinergic neurons impaired odor discrimination learning but inactivation of HDB GABAergic neurons did not. Together, these results suggest that HDB cholinergic and GABAergic neurons are activated differently and may play distinct roles in odor-association learning.

The increased Ca^2+^ signal in HDB cholinergic and GABAergic neurons upon passive odor exposure likely derives from the feedforward connections between olfactory regions and the basal forebrain. Basal forebrain neurons are innervated by several olfactory regions, and c-fos expression in the basal forebrain increases upon odor exposure (Zheng et al., 2018). Moreover, electrical stimulation of the olfactory bulb and cortex modulates neural activity in the HDB (Linster and Hasselmo, 2000). However, whether and how basal forebrain neurons are modulated during passive exposure, especially at the beginning of odor delivery, was previously unknown. Similarly, decreased feedforward input from olfactory regions to the basal forebrain may be responsible for the decreased responses in cholinergic and GABAergic neurons in the late trials of experiments with repeated exposure to odors. Odor habituation is observed in the olfactory system, especially in the olfactory bulb and the piriform cortex (Wilson, 1998; Twick et al., 2014; Ogg et al., 2015; Mignot et al., 2021), and the decreased response in HDB cholinergic and GABAergic neurons after repeated odor exposure may reflect decreased input from these olfactory centers. However, the reverse may also be true. The decreased responses may reflect direct changes in HDB neural activity during passive exposure: HDB neurons may be activated at the beginning of odor exposure as mice pay attention to the novel odor and no longer be activated when the odors become familiar. *Via* feedback connections, the reduction in HDB neural activity may account for odor habituation in olfactory centers such as the olfactory bulb and the piriform cortex. Indeed, it has been reported that basal forebrain neurons can signal novelty (Sun et al., 2019), dishabituate odor responses, and reinstate odor investigation (Ogg et al., 2018).

Higher c-fos levels are observed in basal forebrain cholinergic neurons after olfactory learning (Zheng et al., 2018). Importantly, the basal forebrain neurons are recruited and display higher neural activity during reward association (Devore et al., 2016; Nunez-Parra et al., 2020). Therefore, higher rates of neural activity during odor associative learning may be responsible for the stable responses of HDB cholinergic neurons throughout the go/go task. On the other hand, GABAergic responses decayed rapidly toward the end of the go/go task, indicating that HDB GABAergic neurons may not be activated during reward learning. Thus, the distinct dynamics in cholinergic and GABAergic neurons resulting from different neural activity states may explain why the responses in cholinergic neurons remained stable but the responses in GABAergic neurons decreased over time. Differing levels of activity have been previously reported for basal forebrain cholinergic and GABAergic neurons: whereas cholinergic neurons were consistently excited during reward association in a go/no-go auditory discrimination task, GABAergic neurons exhibited diverse responses (Harrison et al., 2016). However, further investigation is required to examine how HDB cholinergic neurons respond to odor vs. reward, and during which task period these neurons play an important role.

The possibility that the odor response of cholinergic neurons is shaped by attention is further supported by results from the go/no-go experiment, during which these neurons showed divergent responses to the rewarded odor (S+) and the unrewarded odor (S−) after the mice had learned the odor discrimination task. The faster decay of responses during S− trials may reflect reduced attention to the unrewarded cue in proficient mice. Conversely, the cholinergic responses to the S+ and S− were similar during the learning phase, when the mice had not learned to discriminate between the two odors and attention to both stimuli was necessary. Electrophysiology and fiber photometry recordings have shown that odor-evoked responses in the olfactory bulb diverge after learning and that mitral/tufted neurons carry information about odor value (Doucette and Restrepo, 2008; Wang et al., 2019). This neural representation strategy is likely shaped by top-down innervations, such as the noradrenergic (Yamada et al., 2017; Ramirez-Gordillo et al., 2018) and serotonergic (unpublished data) inputs to the olfactory bulb. However, since separated responses were also observed in basal forebrain cholinergic neurons in the present study, it is important to investigate whether and how HDB cholinergic inputs also contribute to odor representation in the olfactory bulb during odor discrimination.

Many studies have investigated the function of HDB cholinergic and GABAergic neurons in olfaction. Selective lesion of cholinergic neurons that project to the OB, inactivation of cholinergic neurons, and pharmacological blockade of acetylcholine receptors all decrease performance in some olfactory behaviors, including olfactory habituation, perceptual generalization, and odor discrimination (Linster et al., 2001; Linster and Cleland, 2002; Smith et al., 2015; Chan et al., 2017; Ross et al., 2019), and activation of the cholinergic system is beneficial for olfactory behaviors (Ma and Luo, 2012; Cho and Linster, 2020; Takahashi et al., 2021). Compared with cholinergic modulation, GABAergic regulation of olfactory behavior has been less studied. Disruption of GABAergic afferents from the basal forebrain impairs habituation/dishabituation behavior (Nunez-Parra et al., 2013) and several studies have implicated basal forebrain GABAergic projections in neural activity and olfactory processing in the olfactory bulb (Hanson et al., 2020; Villar et al., 2021; Zhou and Puche, 2021; De Saint Jan, 2022). Together, these studies indicate that both cholinergic and GABAergic projections are crucial for sensory processing and olfactory learning.

However, in the present study, although we found that cholinergic neurons play an important role in odor discrimination, GABAergic neurons may not be involved in this type of associative learning. HDB GABAergic responses to the S+ and S− did not separate during either the first block or the best block. Furthermore, whereas inactivation of cholinergic neurons impaired odor discrimination, task performance remained high in mice with inactivation of HDB GABAergic neurons. The apparent conflict between our results and those from previous studies is likely due to the different behavioral tasks used. To test the ability to discriminate odors, Nunez-Parra et al. (2013) used a habituation/dishabituation test, which is an instinctual response to odors and does not require an active learning process; we used a go/no-go test, which requires active learning to associate specific odors with the reward. Another potential alternative explanation for the discrepancy is that odor experience during associative learning leads to plastic changes that compensate for the effects caused by the GABAergic neurons. Thus, the HDB GABAergic neurons may be involved in discriminating odors instinctually but not when the discrimination requires associative learning.

In summary, this study demonstrates the distinct dynamics of HDB cholinergic and GABAergic neurons during odor-association learning, and the different roles these two subpopulations play in odor discrimination. These findings are important for understanding the top–down regulation of sensory systems by different subpopulations of basal forebrain neurons.

## Data Availability Statement

The raw data supporting the conclusions of this article will be made available by the authors, without undue reservation.

## Ethics Statement

The animal study was reviewed and approved by Xuzhou Medical University Institutional Animal Care and Use Committee.

## Author Contributions

AL designed the research. PZ and YZ performed the research. PZ, PL, and YZ analyzed the data. DW and AL wrote the article. All authors contributed to the article and approved the submitted version.

## Conflict of Interest

The authors declare that the research was conducted in the absence of any commercial or financial relationships that could be construed as a potential conflict of interest.

## Publisher’s Note

All claims expressed in this article are solely those of the authors and do not necessarily represent those of their affiliated organizations, or those of the publisher, the editors and the reviewers. Any product that may be evaluated in this article, or claim that may be made by its manufacturer, is not guaranteed or endorsed by the publisher.
