# Supplementary material for: The Response Dynamics and Function of Cholinergic and GABAergic Neurons in the Basal Forebrain During Olfactory Learning
Source: Front Cell Neurosci. 2022 Jul 27;16:911439. doi: 10.3389/fncel.2022.911439 (PMC9363711; doi:10.3389/fncel.2022.911439)
Supplement: Supplementary file 1 [file Data_Sheet_1.docx]

Supplementary Material

# Supplementary Figures


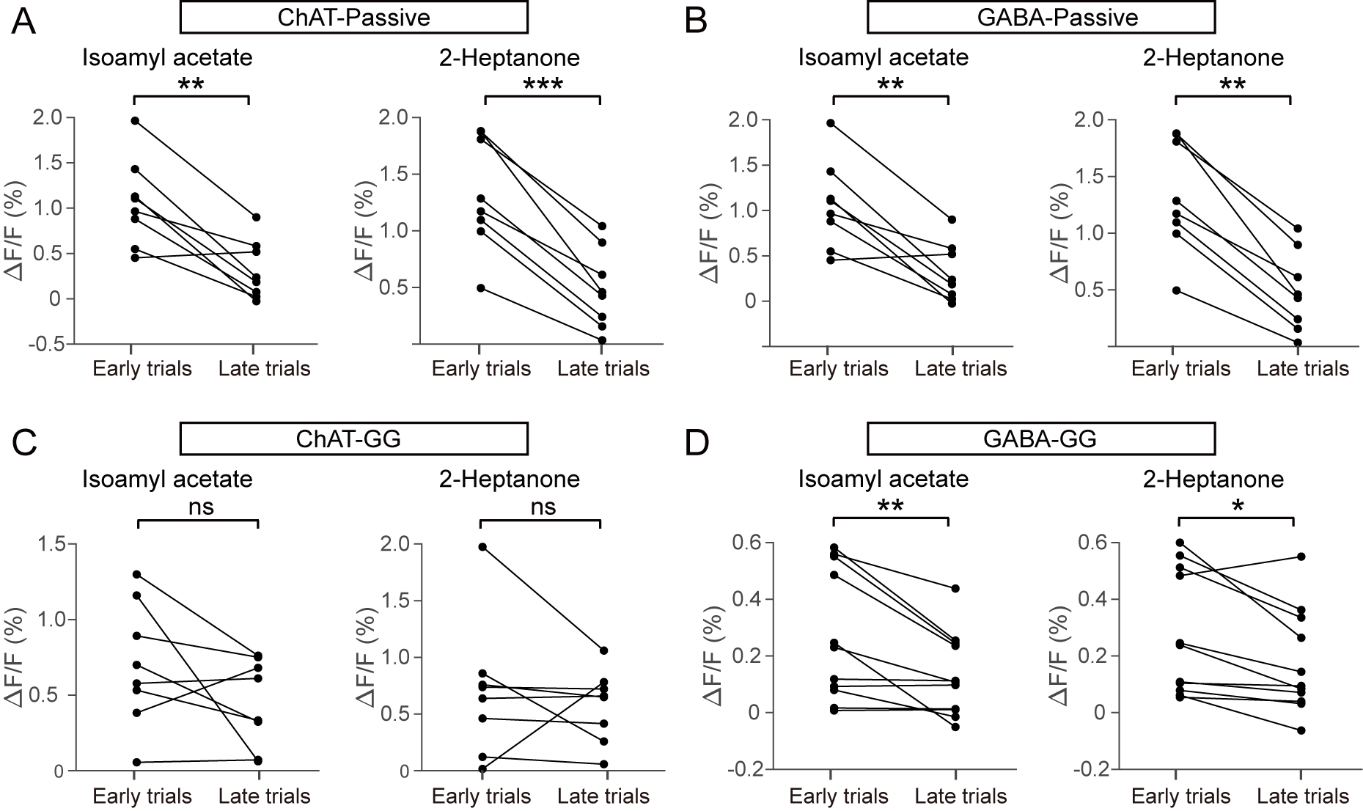


**Supplementary Figure 1.** Comparisons of cholinergic / GABAergic responses in early trials and late trials during passive exposure and the go/go task. (A and B) Both cholinergic (A) and GABAergic (B) responses decreased in late trials (the last 5 trials) compared with early trials (the first 5 trials) during passive exposure. [(A) Isoamyl acetate: paired *t*-test, *t*_(7)_ = 4.83, *P* = 0.0019; 2-Heptanone: paired *t*-test, *t*_(7)_ = 8.24, *P* = 7.57×10^-5^. (B) Isoamyl acetate: paired *t*-test, *t*_(7)_ = 4.56, *P* = 0.0010; 2-Heptanone: paired *t*-test, *t*_(7)_ = 3.33, *P* = 0.0076]. (C and D) Comparisons of cholinergic (C) / GABAergic (D) responses in early trials (the first 10 trials) and late trials (the last 10 trials) during the go/go task. [(C) Isoamyl acetate: paired *t*-test, *t*_(7)_ = 1.66, *P* = 0.14; 2-Heptanone: paired *t*-test, *t*_(7)_ = 0.69, *P* = 0.51. (D) Isoamyl acetate: paired *t*-test, *t*_(10)_ = 3.41, *P* = 0.0066; 2-Heptanone: paired *t*-test, *t*_(10)_ = 3.06, *P* = 0.012]. ns, no significance, ^**^*P* < 0.05, ^**^*P* < 0.01, ^***^*P* < 0.001.

**
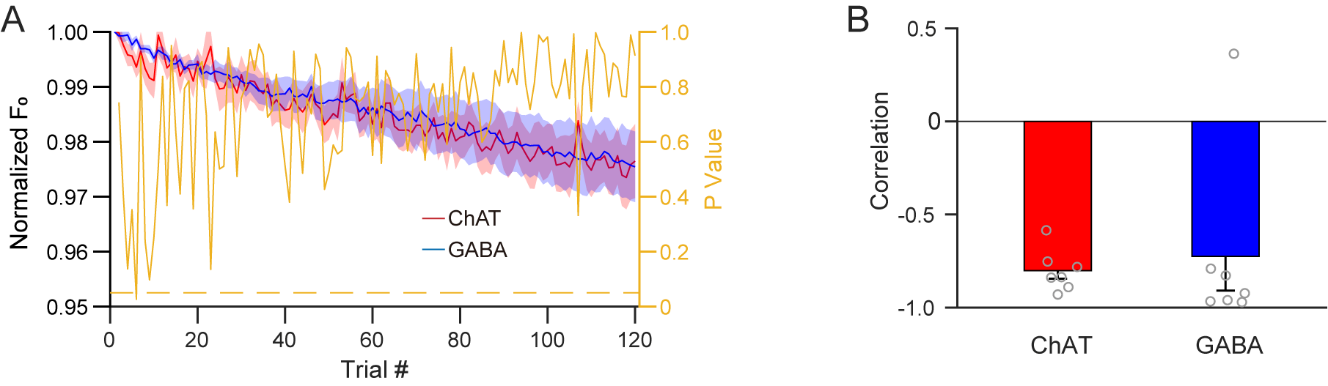
**

**Supplementary Figure 2.** The baseline cholinergic and GABAergic Ca^2+^ fluorescence (averaged Ca^2+^ signals over a 5-s-long time window before the odor presentation) during the 120 trials of the go/go task. (A) Comparison of the normalized Ca^2+^ fluorescence of cholinergic and GABAergic neurons during the go/go task. ChAT: n = 7 animal odor pairs from seven mice. GABA: n = 7 animal odor pairs from seven mice. (B) The correlation between Ca^2+^ fluorescence of cholinergic / GABAergic neurons and the trial numbers. [Wilcoxon signed-rank test, *z* = 1.02, *P* = 0.31].


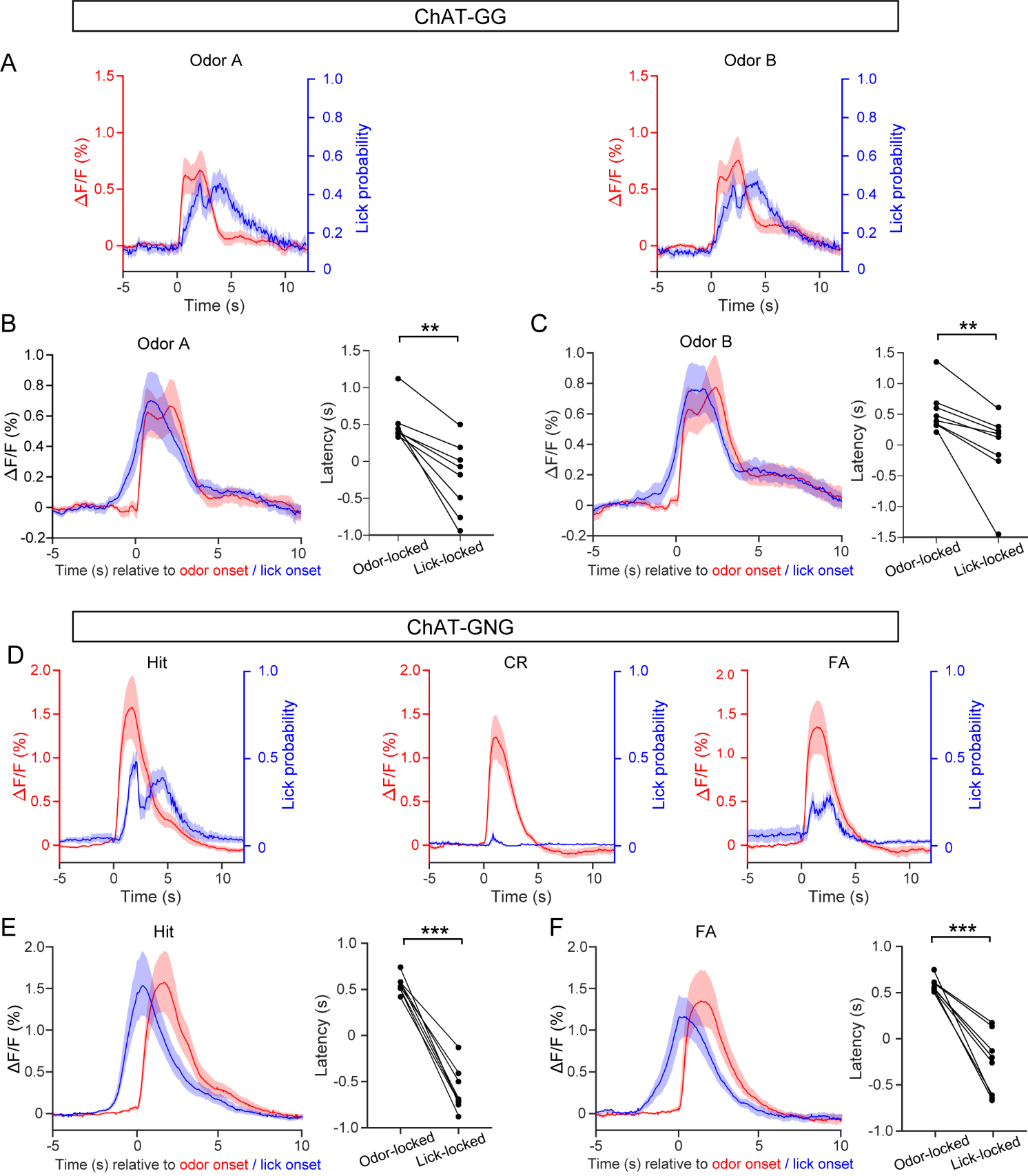


**Supplementary Figure 3.** The lick probability and lick-locked cholinergic responses during the go/go and go/no-go task. (A) The cholinergic responses and lick probability in odor A and odor B trials during the go/go task. (B and C) Left: the cholinergic responses aligned to odor onset (red) and lick onset (blue) in odor A (B) and odor B (C) trials across all animal-odor pairs. Right: The latency from odor onset to 50% peak of the ΔF/F in odor-locked is significantly different from that in lick-locked. [(B) paired *t*-test, *t*_(7)_ = 5.23, *P* = 0.0012; (C) Wilcoxon signed-rank test, *z* = 2.52, *P* = 0.0012]. (D) The cholinergic responses and lick probability in Hit, CR, and FA trials during the go/no-go task. (E and F) Left: the cholinergic responses aligned to odor onset (red) and lick onset (blue) in Hit (E) and FA (F) trials across all animal-odor pairs. Right: The latency from odor onset to 50% peak of the ΔF/F in odor-locked is significantly different from that in lick-locked. [(E) paired *t*-test, *t*_(7)_ = 13.10, *P* = 3.51×10^-6^; (F) paired *t*-test, *t*_(7)_ = 6.95, *P* = 2.21×10^-4^]. ^**^*P* < 0.01, ^***^*P* < 0.001.


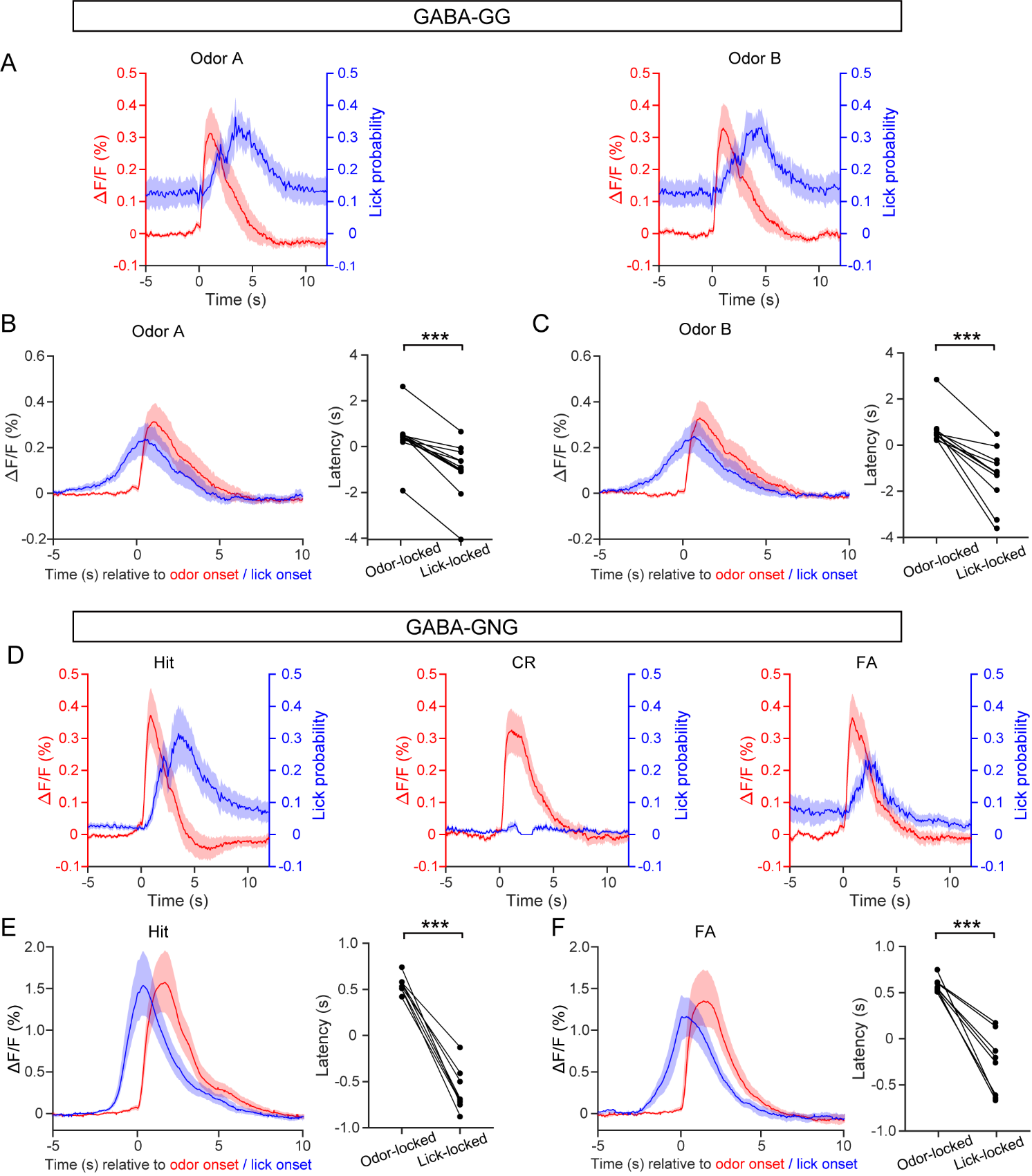


**Supplementary Figure 4.** The lick probability and lick-locked GABAergic responses during the go/go and go/no-go task. (A) The GABAergic responses and lick probability in odor A and odor B trials during the go/go task. (B and C) Left: the GABAergic responses aligned to odor onset (red) and lick onset (blue) in odor A (B) and odor B (C) trials across all animal-odor pairs. Right: The latency from odor onset to 50% peak of the ΔF/F in odor-locked is significantly different from that in lick-locked. [(B) paired *t*-test, *t*_(10)_ = 7.34, *P* = 2.48×10^-5^; (C) paired *t*-test, *t*_(10)_ = 6.06, *P* = 1.22×10^-4^]. (D) The GABAergic responses and lick probability in Hit, CR, and FA trials during the go/no-go task. (E and F) Left: the GABAergic responses aligned to odor onset (red) and lick onset (blue) in Hit (E) and FA (F) trials across all animal-odor pairs. Right: The latency from odor onset to 50% peak of the ΔF/F in odor-locked is significantly different from that in lick-locked. [(E) paired *t*-test, *t*_(12)_ = 16.98, *P* = 9.34×10^-10^; (F) paired *t*-test, *t*_(7)_ = 8.91, *P* = 1.22×10^-6^]. ^***^*P* < 0.001.
